# High-Density Surface EMG-Based Gesture Recognition Using a 3D Convolutional Neural Network

**DOI:** 10.3390/s20041201

**Published:** 2020-02-21

**Authors:** Jiangcheng Chen, Sheng Bi, George Zhang, Guangzhong Cao

**Affiliations:** 1 Shenzhen Academy of Robotics, Shenzhen 518057, China; gqzhang@szarobots.com; 2 School of Computer Science and Engineering, South China University of Technology, Guangzhou 510006, China; 3 Shenzhen Key Laboratory of Electromagnetic Control, Shenzhen University, Shenzhen 518060, China; gzcao@szu.edu.cn

**Keywords:** high-density surface EMG (HD-sEMG), finger gesture recognition, deep learning, convolutional neural network (CNN)

## Abstract

High-density surface electromyography (HD-sEMG) and deep learning technology are becoming increasingly used in gesture recognition. Based on electrode grid data, information can be extracted in the form of images that are generated with instant values of multi-channel sEMG signals. In previous studies, image-based, two-dimensional convolutional neural networks (2D CNNs) have been applied in order to recognize patterns in the electrical activity of muscles from an instantaneous image. However, 2D CNNs with 2D kernels are unable to handle a sequence of images that carry information concerning how the instantaneous image evolves with time. This paper presents a 3D CNN with 3D kernels to capture both spatial and temporal structures from sequential sEMG images and investigates its performance on HD-sEMG-based gesture recognition in comparison to the 2D CNN. Extensive experiments were carried out on two benchmark datasets (i.e., CapgMyo DB-a and CSL-HDEMG). The results show that, where the same network architecture is used, 3D CNN can achieve a better performance than 2D CNN, especially for CSL-HDEMG, which contains the dynamic part of finger movement. For CapgMyo DB-a, the accuracy of 3D CNN was 1% higher than 2D CNN when the recognition window length was equal to 40 ms, and was 1.5% higher when equal to 150 ms. For CSL-HDEMG, the accuracies of 3D CNN were 15.3% and 18.6% higher than 2D CNN when the window length was equal to 40 ms and 150 ms, respectively. Furthermore, 3D CNN achieves a competitive performance in comparison to the baseline methods.

## 1. Introduction

Prosthetic hands that are capable of performing various movements have been, from a mechanical point of view, remarkably improved since the last decade. However, the lack of an effective control interface still prevents its practical application in amputees. Surface electromyography (sEMG) is the non-invasive electrical recording of muscle activity and provides access to neural information associated with human movement. In comparison to touch screens and keyboards, the sEMG-based interface could offer a natural and intuitive way of controlling for disabilities. From its inception until now, the myoelectric prosthesis has been designed with trigger control, proportional control, and pattern recognition-based control [1]. In the pattern recognition-based control approach, a classifier trained with supervised learning was employed to map sEMG activity to one of the predefined classes that correspond to different control commands. In the past decades, many methods have been proposed to design a sEMG pattern recognition-based interface, some of which have achieved high accuracy with many classes in a laboratory environment [2,3,4].

According to the number and the configuration of electrodes, the acquired surface EMG can be divided into sparse multi-channel sEMG and high-density sEMG (HD-sEMG) [5,6,7,8]. With regard to the multi-channel case, great achievements have been made and recognition accuracy has been shown to reach up to 95% in some research [1]. However, the exact positioning of the electrodes (a mostly bipolar configuration) is required to collect the right signals and the signal is considered to represent the activity of the whole muscle. Indeed, the effects of the placement of electrodes were studied in [9]. In other words, the information provided by the sparse multi-channel sEMG is highly dependent on the positioning of electrodes. Another disadvantage of the sparse multi-channel sEMG is that the fault of one channel can cause a reduced performance. In contrast, the HD-sEMG collected by the 2D electrode grids can provide both spatial and temporal information, and have the potential to overcome the aforementioned shortcomings. In previous studies, the concepts of the sEMG image and sEMG map have been proposed for gesture recognition. The sEMG image was spatially composed of instant values of raw HD-sEMG data according to the arrangement of the electrodes, while the sEMG map was composed of values of time windowed features [7,8,10,11,12,13]. At present, the number of electrodes contained in an electrode array could range from 32 to 350, which results in a big dataset with a high sample frequency. While HD-sEMG-based interfaces increase the production cost and computation demand, it is commonly believed that these issues in hardware can be easily resolved with the advancement of sensor devices and special micro-processing technology.

Generally, feature extraction and classifier design are two key factors involved in the development of pattern-recognition-based control interfaces. Thus far, various features have been identified in time, frequency, and time–frequency domains [1,14,15]. Angkoon et al. [14] presented 37 time domain and frequency domain features, and investigated their classification performance. Then, they also evaluated 50 time domain and frequency domain features to improve the robustness of myoelectric pattern recognition [15]. In many studies, multi-features have been used to improve the accuracy of hand gesture/motion classification. For the design of the classifier, the commonly used, conventional classification algorithms include linear discriminant analysis (LDA) [16], hidden Markov models (HMM) [17], support vector machines (SVM) [18], adaptive neuro-fuzzy inference system (ANFIS) [19], and so on. By optimizing the selection of features and classifiers, satisfactory results can be obtained offline. Therefore, the selection of features and classifiers is highly problem-dependent and requires strong professional knowledge, which limits the application of these methods.

Compared to feature engineering and conventional shallow learning models, deep learning models, which contain many hidden layers, can automatically learn a hierarchy of features from raw data and have recently made a huge impact on pattern recognition [20,21]. The deep learning approach is also used in the field of EMG processing [22]. Mukhopadhyay and Samui conducted an empirical exploration on the deep-learning-model-based sEMG signal classification and demonstrated that their methods could outperform other classifiers such as the k-nearest neighbor, random forest, and decision tree [23]. Zhang et al. [24] applied the deep learning method long short-term memory (LSTM) to classify hand gestures by multimodal data collected from the inertial measurement unit, Myo armband (Thalmic Labs Inc.), and pressure sensors. Convolutional neural networks (CNNs) have been successfully used. In the beginning, CNNs were used together with feature pre-extracting (i.e., feature engineering). Allard et al. [25] presented a specific CNN architecture with spectrograms as the input to identify seven hand/wrist gestures. Zhai et al. [26] proposed a CNN-based classifier using a dimension-reduced spectrogram as the input and updated itself to maintain its performance over a long time. In contrast to using the pre-extracted sEMG features as the input, Geng et al. [11] found that there were patterns inside instantaneous HD-sEMG images (formed with raw data) and presented a CNN model to recognize the gestures with instantaneous HD-sEMG images. They later proposed a hybrid CNN and RNN (recurrent neural network) architecture to take advantage of the time series information [27]. Inspired by the correlation between certain muscles and each specific gesture, they also proposed a two-stage multi-stream CNN to enhance recognition accuracy [28]. This method decomposes the original sEMG image into many equal-sized streams and independently learns representative features by 2D CNN.

In summary, HD-sEMG signals that carry both spatial and temporal information of muscle activity and employ CNNs as classifiers are becoming increasingly studied in developing sEMG-based interfaces. However, as a class of deep models, CNNs with 2D convolution kernels are limited to handling a single image that only carries spatial information. In contrast, 3D CNN, with 3D convolution kernels, is capable of extracting features from both spatial and temporal dimensions. The first 3D CNN was proposed by Ji et al. [29] to analyze video data, and achieved a superior performance in comparison to the baseline methods. Besides this, Al-Hammadi et al. [30] employed transfer learning of a 3D CNN for hand gesture recognition by using video data and achieved excellent accuracy. In fact, HD-sEMG signals are sequential data like video data. Motivated by this, we investigated a CNN with 3D convolution kernels for HD-sEMG pattern recognition in this paper. To the best of our knowledge, this is the first comparative study of 2D CNN and 3D CNN in HD-sEMG-based gesture recognition.

The rest of the paper is organized as follows: The materials and methods including the data used in this paper, the construction of the 3D CNN, and the experiments of HD-sEMG-based finger gesture recognition are described in Section 2. The experimental results are presented and discussed in Section 3. The paper is concluded in Section 4.

## 2. Materials and Methods

### 2.1. Data and Pre-Processing

**Figure 1:**
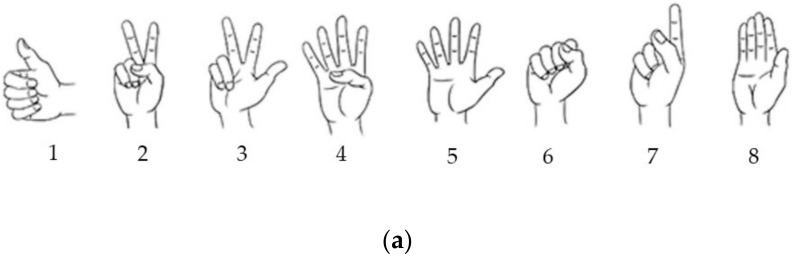

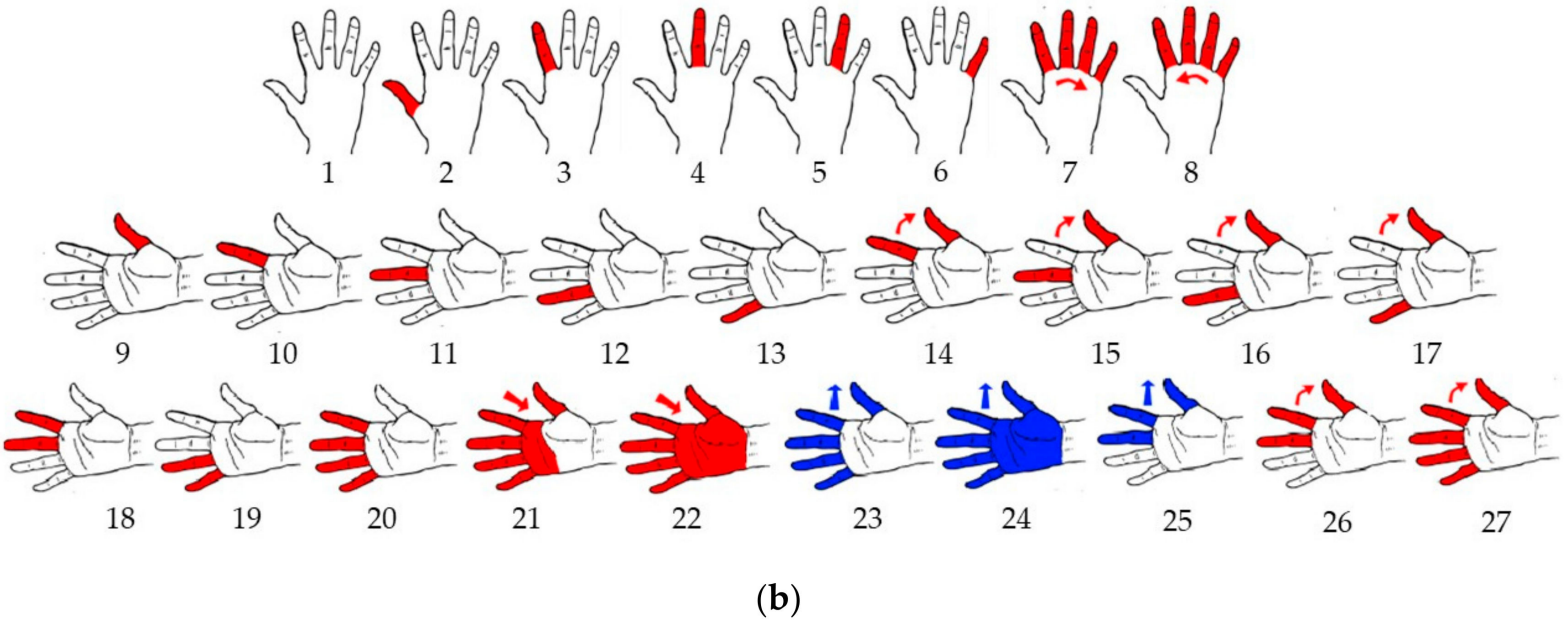
Iconic illustrations of eight gestures contained in CapgMyo DB-a and 27 gestures contained in SCL-HDEMG. (**a**) Eight finger gestures. These are (1) thumb up, (2) extension of index and middle finger while flexing the others, (3) flexion of the ring and little fingers while extending the others, (4) thumb opposing base of the little finger, (5) abduction of the fingers, (6) fingers flexed together in a fist, (7) pointing index, and (8) adduction of extended fingers, respectively. (**b**) Twenty-seven finger gestures. (1) idle gesture, (2–6) tap once with the finger shown in red, (7–8) four fingers are tapped sequentially in the direction of the arrow, (9–13) bending of the finger, (14–17) pinch grips with the thumb, (18–20) bending of two or four fingers, (21) making a fist without applying force, (22) making a fist with force, (23–25) stretching out the fingers marked in blue, and (26–27) pressing the marked fingers against the thumb.

CapgMyo and CSL-HDEMG are two HD-sEMG benchmark databases and were utilized in this study. Both of them are available online [11,12]. For CapgMyo, we downloaded the sub-database named DB-a, which contains eight isometric and isotonic finger gestures obtained from 18 healthy able-bodied subjects. The eight finger gestures are shown in Figure 1a. Each subject performed 10 trials for each gesture. Each gesture was held for 3 to 10 seconds. The HD-sEMG signals in CapgMyo DB-a were recorded with an 8 × 16 electrode grid wrapped around the right forearm. The 128-channel signals were band-pass filtered at 20–380 Hz and sampled at 1000 Hz. The CSL-HDEMG database contained 27 finger gestures obtained from five subjects. The 27 finger gestures covered the extension and flexion of individual fingers and incorporated some typical gestures that might be used in human–computer interaction. These were organized in three sets in [12], which were tapping gestures, bending gestures, and multi-finger gestures, respectively. The signals were bipolarly recorded by a 7 × 24 electrode grid that led to a total of 168 channels of usable data by removing the channels of data from the difference of the last electrode in one column and the first electrode in the next column. The HD-sEMG signals in CSL-HDEMG were sampled at 2048 Hz and each trial was recorded for three seconds. Each subject was asked to perform one gesture within a time interval of three seconds. Each subject recorded five sessions and performed 10 trials for each gesture in every session. The gestures in CSL-HDEMG and their indexes are shown in Figure 1b.

**Figure 2:**
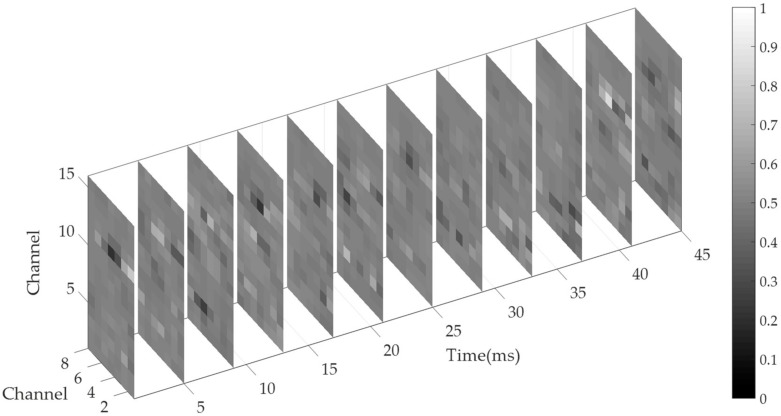
The partial of sequential surface electromyography (sEMG) images obtained from one selected trial of CapgMyo DB-a. Signals were sampled in time at 1000 frames/s. Twelve instantaneous images (out of 45, interval = 4 ms) are shown in the figure. Each pixel of the image represents one channel. The brighter the pixel, the stronger the electrical activity of the channel.

For the purposes of this study, first, the sequential sEMG images should be obtained. For CapgMyo DB-a, there were two datasets: the preprocessed dataset and the raw dataset. We selected the preprocessed dataset in which the power-line interference has been removed and the values of the signal have been normalized to [−1, 1]. Furthermore, the sEMG signals of each trial in the preprocessed dataset contain 1000 frames of data that correspond to the static part of finger movement. In other words, for each trial, the middle one-second data have been segmented. Therefore, in our work, we only transformed the values of the signals to [0, 1] linearly and reshaped the data of each frame to form an 8 × 16 sEMG image. As a result, 1000 sequential sEMG images were generated for each finger gesture recording per subject. Figure 2 shows the partial, sequential sEMG images obtained from one selected trial. From the figure, we can see that the instantaneous image evolved with time, although they corresponded to a static part of the finger movement.

For CSL-HDEMG, more processing was required to obtain the sequential sEMG images. Like the experiments of Amma et al. [12], the sEMG signals were first filtered by a zero-lag fourth-order Butterworth filter with a pass-band of 20–400 Hz to remove signal artifacts. Then, full-wave rectification, followed by a low-pass zero-lag Butterworth filter with a 4 Hz cutoff frequency were applied on the denoised signals and the time series of the intensities were acquired [31]. Next, the thresholding approach, on a sliding window without overlap, was applied on the time series of the intensity to search for the segment containing activity. The length of the sliding window was set to 150 sampling points (73.2 ms) [12], resulting in the length of the segmented muscle activity being a multiple of 150. Instead of calculating the root-mean-square (RMS) in the work by Amma et al. [12], the average intensity value on every window was calculated for each channel and the average of the summarized values of all channels per window was chosen as the threshold for the segmentation in our work. Based on the above process, the activity segments for all 168-channel sEMG signals in every trial were identified. Finally, the values of the denoised signals were transformed to linear and sequential sEMG images [0,1], corresponding to the 27 gestures that were generated for each trial. The segmented sequential sEMG images included continuous finger movement instead of just static movement, like that seen in CapgMyo DB-a.

### 2.2. Construction of 3D Convolutional Neural Network

#### 2.2.1. 3D Convolution

Encouraged by the achievements in the field of image recognition, CNNs have been extended to be three-dimensional using 3D kernels to make them suitable for video sequences or volumetric medical imaging data [29,32]. In 2D CNNs, units in a convolution layer are organized in planes within which all the units share the same set of weights to perform 2D convolution on the local neighborhood of a feature map in the previous layer. The area of the local neighborhood is called the receptive field of the unit and its size is the size of a 2D convolution kernel. A complete convolution layer is composed of several planes and the outputs of each plane generate a feature map. Compared to 2D CNN, 3D convolution is achieved in 3D CNN by convolving a 3D kernel to the cube formed by stacking multiple contiguous frames together. The 3D convolution on a cube will produce another cube. As a result, 3D CNNs perform convolution spatiotemporally and capture both the spatial and temporal structures, while 2D CNNs perform convolution spatially and only capture spatial information. Formally, the value at position $\left(x,y,z\right)$ in the jth cube in the ith layer is given by

(1) $$\upsilon_{ij}^{xyz}=f\left(b_{ij}+\underset{m}{\sum }\overset{P_{i}-1}{\underset{p=0}{\sum }}\overset{Q_{i}-1}{\underset{q=0}{\sum }}\overset{R_{i}-1}{\underset{r=0}{\sum }}\omega_{ijm}^{pqr}\upsilon_{\left(i-1\right)m}^{\left(x+p\right)\left(y+q\right)\left(z+r\right)}\right)$$

where $P_{i}$,$\;Q_{i}$, and $R_{i}$ are the height, width, and length of the kernel, respectively. m is the index of the set of cubes in the previous layer. $\omega_{ijk}^{pqr}$ is the $\left(p,q,r\right)\mathrm{th}$ of the kernel connected to the *k*th cube in the previous layer. $\;\upsilon_{ij}^{xyz}$ designates the output of the jth cube in the ith layer at the $\left(x,y,z\right)$ position. $b_{ij}$ is the bias. $f\left(\cdot \right)$ represents the activation function, which in most cases is a sigmoid function, tan hyperbolic, or rectified linear unit (RELU).

#### 2.2.2. 3D CNN Architecture

In this section, we present an ordinary 3D CNN architecture that was used in this paper. While several studies on 3D CNNs have been conducted, there has been no rigorous theoretical analysis in designing the architecture of the network. Usually the depth and the kernel size are task-dependent. The first 3D CNN model, proposed by Ji et al. [29], consisted of one hardwired layer, three convolution layers, two sub-sampling layers, and one fully connected layer. Tran et al. [33] proposed a 3D CNN architecture with eight convolutions, five max-pooling, and two fully connected layers that were designed to learn spatiotemporal features from a video dataset. Their findings suggest that a homogeneous architecture with small 3 × 3 × 3 convolution kernels could achieve the best performance. A 3 × 3 kernel is also employed for the 2D CNN model, which is often used for gesture recognition based on instantaneous surface EMG images [11].

**Figure 3:**
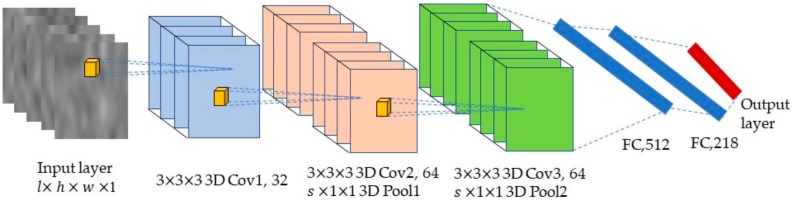
The architecture of the proposed 3D convolutional neural network. The network consists of an input layer, three convolutional layers, two pooling layers, two fully connected layers, and an output layer. The input of the network is a clip of a sequence of sEMG images and is expressed as l × h × w × 1, where l is the number of continuous sEMG images, h and w are the height and width of each image, respectively, and 1 indicates that the sEMG images are grayscale images. The outputs of the network are the classes of the finger gestures.

The finally determined 3D CNN architecture is illustrated in Figure 3, based on previous studies, and was the purpose of our exploration. The network contained one input layer, three 3D convolutional layers, two pooling layers, two fully connected layers, and ended with a G-way fully connected layer and a *softmax* function, where G is the number of gestures to be classified. The input layer is the first layer and specifies the size of the input. We refer to the input cube with a size of l × h × w × c, where l is the number of frames, c is the number of channels (c=1 due to the grayscale of sEMG image and the c will be omitted in the following text), and h and w are the height and width of each image, respectively. The 3D kernel size is indicated by d × k × k, where d is the temporal depth and k is the spatial size. The number of kernels in the three convolutional layers were 32, 64, and 64, respectively. The size of all kernels was set to 3 × 3 × 3 and the convolution stride was fixed to 1 × 1 × 1. Zero padding with a size of 0 × 1 × 1 was applied for the second and third convolutional layers, followed by a pooling layer with a size of s × 1 × 1, where the choice of s was related to the sampling rate of the sEMG signals in this study. This meant that sub-sampling was only performed temporally. The two fully connected layers consisted of 512 and 128 neurons, respectively. The rectified linear unit (RELU) activation function was applied for all the hidden layers.

#### 2.2.3. Network Training

The proposed 3D CNN was implemented based on the platform of Keras 2.3.0, which is a high-level neural network application programming interface (API), written in Python and capable of running on top of TensorFlow. The workstation we employed included a Nvidia GTX 1080 Ti GPU and one Intel Core i7 CPU. The stochastic gradient descent algorithm was used to update the parameters. The initial learning rate was set to 0.1 and was divided by two if there was no loss in improvement after 10 iterations. During the training stage, the following cross-entropy loss function was utilized to optimize the output

(2) $$\mathrm{Loss}=\;-\frac{1}{n}\overset{n}{\underset{i=1}{\sum }}y_{i}\log{\bar{y}}_{i}+\left(1-y_{i}\right)\log\left(1-{\bar{y}}_{i}\right)$$

where $y_{i}$ and ${\bar{y}}_{i}$ are the sample labels and the actual output, respectively, and n is the number of input samples. In addition, batch normalization before each non-linearity layer and dropout with a probability of 0.5 on the two fully connected layers were employed to optimize the network, where the former can accelerate the network training and the latter can prevent the network from over-fitting [34,35].

### 2.3. Majority Voting

With the trained 3D network, the patterns for each input cube could be recognized. As described above, the length of the cube could range from one to many frames. In order to satisfy the real-time usage constraints, the recognition time should be shorter than 300 ms [27]. In fact, multiple input cubes could be generated in a 300 ms time period. Therefore, simple majority voting over multiple consecutive recognition results could be used to enhance the recognition accuracy. The voting window needed to be less than 300 ms. Considering that, in a voting window, the recognition results of a neural network for consecutive cubes are $M_{i}\;\left(i=1,2\ldots ,n_{c}\right)$ and the target gesture is T, then the recognition accuracy P can be calculated as

(3) $$P=\;\frac{m}{n_{c}}\times 100\%\;\;$$

where m is the number of recognition results that satisfy $M_{i}=T$. What should be emphasized here is that the unit of the voting window in this work is in ms. In other words, if the length of the input cube, which was equal to the length of the sliding window for segmentation, was $t_{\mathrm{cube}}$ ms and there were $n_{c}$ consecutive cubes in a voting window, then the length of the voting window was defined as $n_{c}\cdot t_{\mathrm{cube}}$ ms.

### 2.4. Experiments

The proposed model was evaluated on the two benchmark databases described in Section 2.1. The CapgMyo DB-a database was divided into two parts of the same size for training and testing, as described in [11]. The sequential sEMG images of each trial were split into small segments using the sliding window strategy and a number marked each sEMG cube as one of the gestures. The cube size was l × 8 × 16. Considering that a large number of labeled samples were required for network learning, the sliding window using the overlap method for EEG data augmentation was applied on CapgMyo DB-a [36]. What needs to be emphasized here is that only the data from the training part adopted the data augmentation strategy (with a stride two frames) during segmentation. Additionally, the size of the pooling layer was set to 2 × 1 × 1. For the CSL-HDEMG database, the leave-one-out cross-validation scheme was performed in each session on every subject at the same time in [12], in which each of the 10 trials served as testing data while the remaining nine trials constituted the training data. Similarly, the sequential sEMG images of each trial were split into small clips by sliding windows. The cube size was l × 7 × 24 and the size of the pooling layer was set to 4 × 1 × 1. In the experiments, we investigated the recognition accuracies with different lengths l of the sliding window. For comparison purposes, we constructed a 2D CNN to perform recognition after supervised learning. As far as we know, except for the form of convolutions, the predictive capability of the model was also related to the structure and depth of the network. Therefore, in order to make a fair and effective comparison, the three 3D convolutional layers in the proposed network were replaced by three 2D convolutional layers. The temporal dimensions of the kernels for convolution, padding, and pooling were removed, while the size of the spatial dimensions remained the same. Together with the majority voting strategy over consecutive frames, the recognition accuracies of 2D CNNs, with different voting window lengths, were evaluated on the two databases. Furthermore, the statistics of recognition accuracies were compared with the results from previous studies.

## 3. Results and Discussion

**Figure 4:**
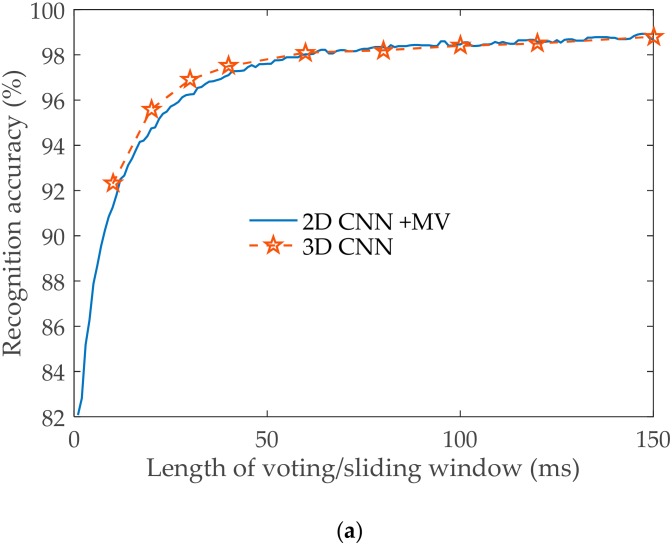

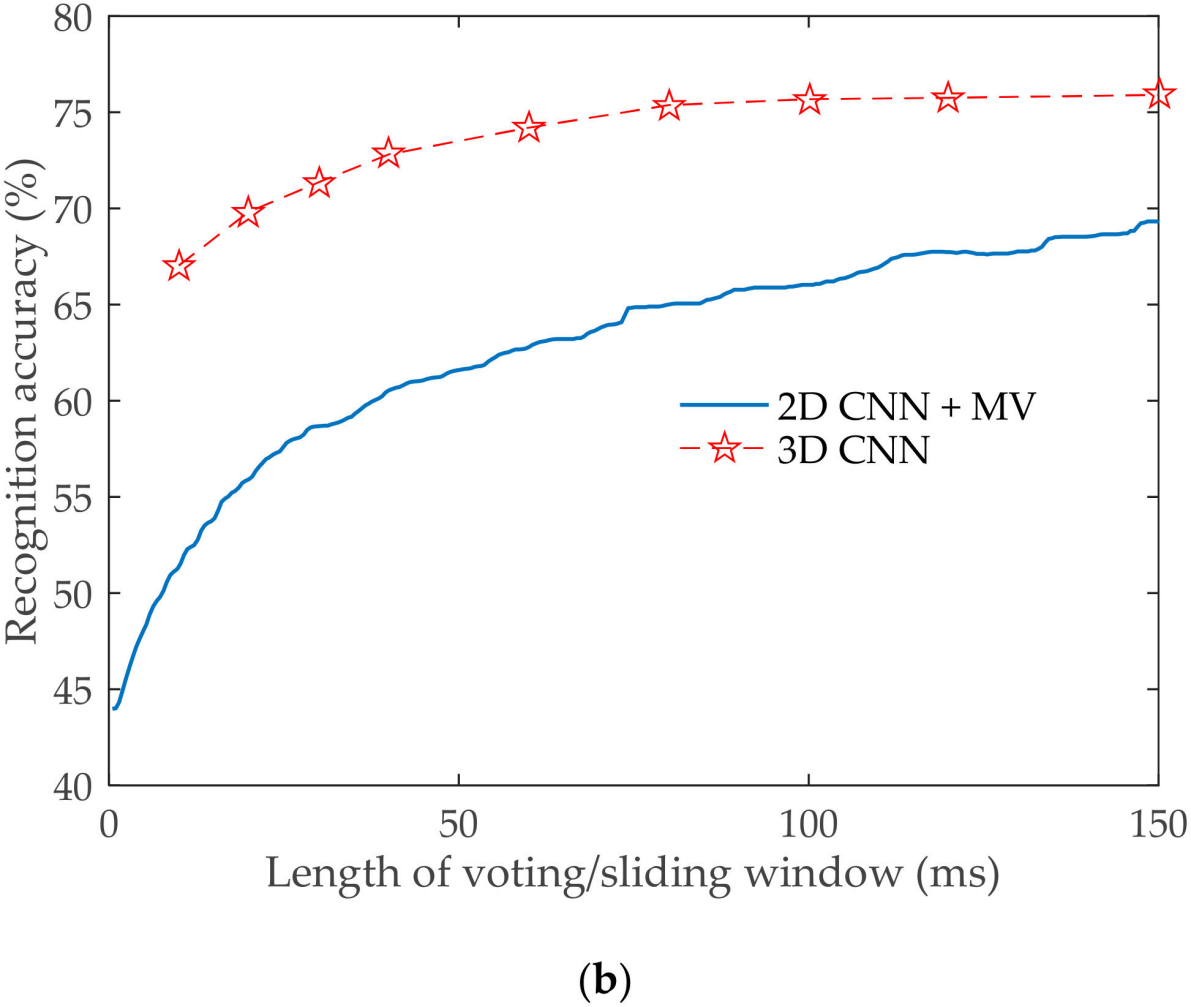
The comparation of recognition accuracies between 3D CNN over different sliding windows and 2D CNNs over different voting windows. (**a**) Recognition accuracies over the first 6 subjects of the CapgMyo DB-a database. (**b**) Recognition accuracies of subject1–session1 of the CSL-HDEMG database.

Figure 4 shows the comparison of recognition accuracies between the 3D and 2D CNNs proposed in this paper; Figure 4a shows the statistical results from the CapgMyo DB-a database, while Figure 4b shows the statistical results from the CSL-HDEMG database. In Figure 4a, the blue solid line represents the recognition accuracies over the first six subjects by using 2D CNN, together with the majority voting (2D CNN + MV) method with respect to different lengths of voting windows. It can be seen that the recognition accuracy increases with increasing lengths of voting windows, where the accuracies corresponding to the lengths of 10 ms and 150 ms were 91.3% and 98.7%, respectively. The pentagram connection indicates the recognition accuracies over the first six subjects by using 3D CNN with different lengths l of the sliding window for segmentation, where the lengths at the location of the pentagrams were 10, 20, 30, 40, 60, 80, 100, 120, and 150 frames, respectively. The accuracies corresponding to the lengths of 10 and 150 were 92.3% and 98.9%, respectively. It was found that the recognition accuracy of the 3D CNN was higher than the 2D CNN + MV when the length of the sliding/voting window was less than 60, and was basically the same when the length of the window was higher. In Figure 4b, the blue solid line represents the recognition accuracies of subject1–session1 by using 2D CNN + MV, while the pentagram connection represents the recognition accuracies by using 3D CNN. Figure 4b shows that the accuracy obtained by both methods increased with an increase in the length of the window, which was the same as the case using CapgMyo DB-a. It was also found that the recognition accuracies by using 3D CNN were significantly higher than by using 2D CNN + MV, in comparison to the case using CapgMyo DB-a. The accuracies corresponding to the lengths of 10 ms (20 frames) and 150 ms (307 frames) were 51.4% and 70.4% with 2D CNN + MV and were 67.0% and 75.9% with 3D CNN.

In contrast, the improvement in the accuracy of CSL-HDEMG was significantly greater than that of CapgMyo DB-a. This is partly because the improvement space is limited due to CapgMyo DB-a being close to saturation, but the most important reason is that only the static part of the finger movement is included for CapgMyo DB-a, while the dynamic part is also included in CSL-HDEMG. Therefore, the 3D CNN method, capable of extracting dynamic change information, has a great advantage. It can also be seen from Figure 4 that the increase in accuracy was not obvious after a certain length for the 3D CNN. Therefore, it is not worth selecting a long length for the input cube of the 3D CNN because with the increase in length, the number of weights needing to be learnt increases rapidly, leading to a higher computation cost. The situation in this paper is shown in Table 1. In contrast, the number of weights needing to be learnt for the 2D CNN, in the cases of CapgMyo DB-a and CSL-HDEMG, were $2.90\times 10^{6}$ and $3.77\times 10^{6}$, respectively.

Based on the above findings, we can conclude that, in the case of the same network architecture, 3D convolution can achieve better performance than the combination of the 2D convolution and majority voting, especially for the sEMG data, which contain distinctly dynamic processes. However, it is necessary to make a trade-off between accuracy and computation load. The computation load is directly related to the length of the window for segmentation. Therefore, we will investigate the combined use of the 3D CNN with a short length and the majority voting strategy (3D CNN + MV) below. From the perspective of computation load, the lengths of the sliding windows chosen in this work for segmentation were 10 and 20 frames for CapgMyo DB-a and CSL-HDEMG, respectively. Table 2 shows the recognition accuracies over the entire database by using 3D CNN + MV. The results of 2D CNN + MV are also presented in the table. First, it shows that the recognition accuracy of the 3D CNN + MV method was higher than the 2D CNN + MV method. For CapgMyo DB-a, the accuracy of 3D CNN + MV corresponded to the voting length of 40 ms and 150 ms and were 95.5% and 98.6%, respectively, which was lower than the results (99.0% and 99.6%, respectively) presented in [11]. However, for CSL-HDEMG, the accuracy of 3D CNN + MV corresponded to a voting length of 150 ms and was 90.7%, which was higher than the 89.3% presented in [11]. This further confirms the advantages of 3D CNN in the pattern recognition of EMG signals with dynamic processes.

**Figure 5:**
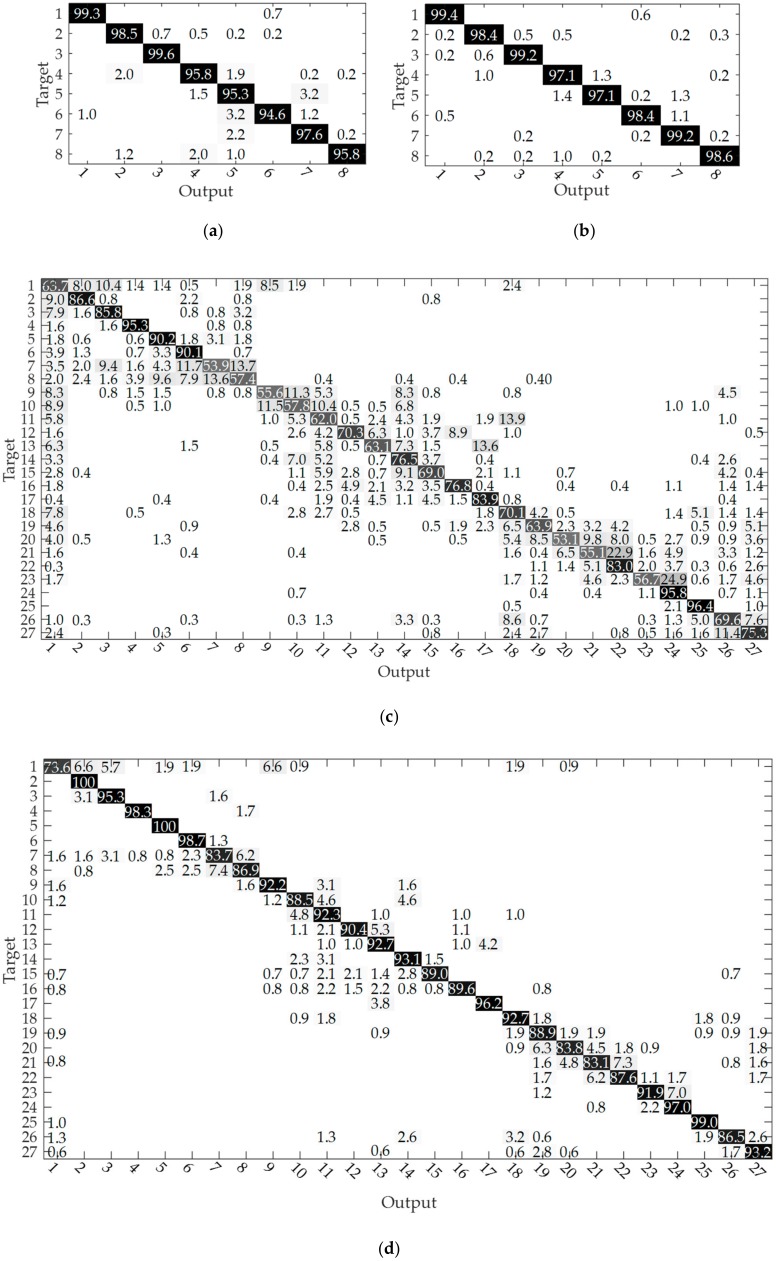
Accumulated confusion matrixes of each method for both databases corresponding to the voting length 150 ms. The higher the percentage value, the darker the color. (**a**) The case of 2D CNN + MV for CapgMyo DB-a. (**b**) The case of 3D CNN + MV for CapgMyo DB-a. (**c**) The case of 2D CNN + MV for CSL-HDEMG. (**d**) The case of 3D CNN + MV for CSL-HDEMG.

The recognition accuracies in Table 2 are a global measure. In order to compare the classification rate of individual gestures between different approaches, and considering the imbalance in the dataset results from the activity interval segmentations, the four accumulated confusion matrixes, corresponding to the voting length of 150 ms (in Table 2) over all recognition results, were calculated as examples of all the approaches taken, as shown in Figure 5. The values in the matrixes were rounded. From Figure 5a, we can find that the lowest classification accuracy is gesture 6, which is often confused with gesture 5. However, in the case of using the 3D CNN + MV method, the accuracy rate of gesture 6 increased to 98.4% and this gesture showed no confusion with gesture 5. Furthermore, the accuracy rates for gestures 4–8 all increased, while gestures 1–3 basically remained the same. From Figure 5c,d, we can see that there were many gestures with accuracy rates between 50% and 60% using the 2D CNN + MV method, while the lowest individual accuracy rate was 73.6% (gesture 1, which is often confused with gestures 2, 3, and 9) by using the 3D CNN + MV method. We can also see from the number distributions of the two figures that confusion occurred more frequently with 2D CNN + MV than with 3D CNN + MV. The above findings confirm the better performance of the 3D CNN.

**Figure 6:**
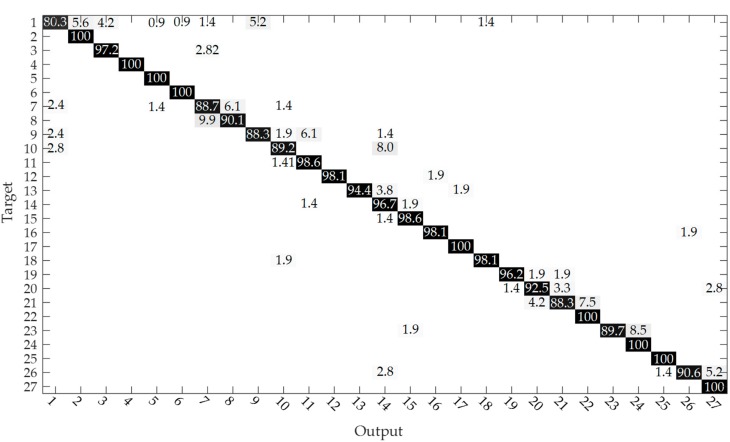
Accumulated confusion matrix over all subjects and sessions. The higher the percentage value, the darker the color.

We also counted the results of simple majority voting over the entire segment of each trial for CSL-HDEMG by using the 3D CNN + MV method. The accuracy reached 95.3%, which was higher than the 90.4% presented in [12], and close to the 96.7% presented in [11] and 96.1% presented in [25]. Figure 6 shows the accumulated confusion matrix over all subjects and sessions by using 3D CNN + MV with an input of size 20 × 7 × 24 and majority voting over the entire segment of each trial. From the figure, we can see that gesture 1 (i.e., idle gesture) is the most confused gesture due to its confusion with gestures 2, 3, and 9. This is consistent with the results in [12]. Gesture 1 had the lowest rate at only 80.3%, while some gestures had 100% accuracies. Gestures 7 and 8 were often confused, in addition to gestures 23 and 24. We could also see that gesture 21 was frequently confused with gestures 20 and 22. This may be due to the similarity between these confusing gestures such as gestures 7 and 8, gestures 23 and 24 and gestures 21 and 22.

## 4. Conclusions

In this study, we constructed a 3D convolutional neural network (3D CNN) to perform high-density surface EMG (HD-sEMG) based gesture recognition. Based on the two benchmark datasets (i.e., CapgMyo DB-a and CSL-HDEMG), the performance of 3D CNNs as well as its comparation to 2D CNN were thoroughly investigated. From the above experimental studies and analysis, we can obtain the following conclusions. First, in the case of the same network architecture, 3D convolution can achieve a better performance than the combination of 2D convolution and majority voting, especially for the sEMG data that contain the dynamic part of the finger movement. This benefit comes from the capability of 3D CNN to capture both the temporal and spatial information of muscle activity from high-density surface EMG signals collected by a grid of electrodes. Second, when the length of the input clip increases, the time taken to learn and recognize the 3D CNN increases quite rapidly. Therefore, a trade-off between recognition accuracy and computation load should be made. Additionally, by using the 3D CNN, together with the majority voting strategy, a competitive performance, in comparison to the baseline methods, can be achieved. In summary, HD-sEMG based gesture recognition, by using 3D CNN, could be a promising solution to develop muscle interfacing for prosthetic control. In addition, the 3D CNN architecture used in this work is relatively simple in comparison to the deep neural network used in other studies. In future research, we will try to explore newer and deeper neural network architectures and add 3D convolution operations to further improve the accuracy of HD-sEMG based gesture recognition.

## Figures and Tables

**Table 1:** The number of weights needing to be learnt over different lengths l of the sliding window for segmentation (i.e., the length of the input cube).

$l\left(\mathrm{ms}\right)$	10	20	30	40	60	80	100	120	150
**Number** $\left(\times 10^{6}\right)$ ^1^	3.08	8.71	17.11	22.75	36.79	50.80	64.86	78.87	101.29
**Number** $\left(\times 10^{6}\right)$ ^2^	4.05	7.88	15.36	19.20	26.88	38.18	49.50	57.18	72.31

^1^ In the case of the CapgMyo DB-a database. ^2^ In the case of the CSL-HDEMG database.

**Table 2:** The recognition accuracies over the entire database with different lengths $l_{V}$ of voting windows.

$l_{V}\;(\mathrm{ms})$	40	50	60	70	80	90	100	110	120	130	140	150
2D CNN + MV (%) ^1^	94.5	95.3	96.0	96.2	96.4	96.4	99.6	96.7	96.7	96.8	96.8	97.1
3D CNN + MV (%) ^1^	95.5	96.7	97.2	97.8	97.9	98.0	98.3	98.3	98.4	98.4	98.6	98.6
2D CNN + MV (%) ^2^	61.7	62.7	64.1	64.9	65.8	67.1	67.7	67.8	69.6	69.6	71.0	72.1
3D CNN + MV (%) ^2^	77.0	79.1	81.1	83.4	84.6	84.7	86.5	86.9	87.9	89.5	90.6	90.7

^1^ In the case of CapgMyo DB-a database. ^2^ In the case of CSL-HDEMG database.
